# A Real-Time Brain-Machine Interface Combining Motor Target and Trajectory Intent Using an Optimal Feedback Control Design

**DOI:** 10.1371/journal.pone.0059049

**Published:** 2013-04-10

**Authors:** Maryam M. Shanechi, Ziv M. Williams, Gregory W. Wornell, Rollin C. Hu, Marissa Powers, Emery N. Brown

**Affiliations:** 1 School of Electrical and Computer Engineering, Cornell University, Ithaca, New York, United States of America; 2 Department of Electrical Engineering and Computer Science, University of California, Berkeley, California, United States of America; 3 Department of Electrical Engineering and Computer Science, Massachusetts Institute of Technology, Cambridge, Massachusetts, United States of America; 4 Department of Neurosurgery, Massachusetts General Hospital, Boston, Massachusetts, United States of America; 5 Harvard Medical School, Boston, Massachusetts, United States of America; 6 Department of Anesthesia, Critical Care and Pain Medicine, Massachusetts General Hospital, Boston, Massachusetts, United States of America; 7 Department of Brain and Cognitive Sciences, Massachusetts Institute of Technology, Cambridge, Massachusetts, United States of America; 8 Institute for Medicine, Engineering and Science, Massachusetts Institute of Technology, Cambridge, Massachusetts, United States of America; University of Maryland, College Park, United States of America

## Abstract

Real-time brain-machine interfaces (BMI) have focused on either estimating the continuous movement trajectory or target intent. However, natural movement often incorporates both. Additionally, BMIs can be modeled as a feedback control system in which the subject modulates the neural activity to move the prosthetic device towards a desired target while receiving real-time sensory feedback of the state of the movement. We develop a novel real-time BMI using an optimal feedback control design that jointly estimates the movement target and trajectory of monkeys in two stages. First, the target is decoded from neural spiking activity before movement initiation. Second, the trajectory is decoded by combining the decoded target with the peri-movement spiking activity using an optimal feedback control design. This design exploits a recursive Bayesian decoder that uses an optimal feedback control model of the sensorimotor system to take into account the intended target location and the sensory feedback in its trajectory estimation from spiking activity. The real-time BMI processes the spiking activity directly using point process modeling. We implement the BMI in experiments consisting of an instructed-delay center-out task in which monkeys are presented with a target location on the screen during a delay period and then have to move a cursor to it without touching the incorrect targets. We show that the two-stage BMI performs more accurately than either stage alone. Correct target prediction can compensate for inaccurate trajectory estimation and vice versa. The optimal feedback control design also results in trajectories that are smoother and have lower estimation error. The two-stage decoder also performs better than linear regression approaches in offline cross-validation analyses. Our results demonstrate the advantage of a BMI design that jointly estimates the target and trajectory of movement and more closely mimics the sensorimotor control system.

## Introduction

There has been a large body of work in the past decade on real-time brain-machine interfaces (BMI) demonstrating that neural signals from the motor cortical areas can be used to control computer cursors or robotic arms in human and non-human primates [1]–[22]. One type of such BMIs, which comprises most of this work, aims to estimate a continuous trajectory—for example the position of a computer cursor on the screen moving towards a visual target [1]–[14], [19]. Recent efforts with this type of BMIs have demonstrated the ability to estimate continuous movement from motor cortical activity. The other type of BMIs aim to predict a desired discrete target without estimating the corresponding desired trajectory towards it [15], [16] and are valuable for purposes such as typing on a keyboard. Recently, we designed another type of BMI for sequential motor function that can concurrently decode the full motor sequence before movement initiation [20].

The successful real-time attempts at individual decoding of the continuous trajectory or the target of movement motivate the development of a new type of real-time BMIs that aim to estimate jointly both the trajectory of the movement and the intended target. This approach is justified by two main reasons. First, the activity in the motor cortical areas has been shown to be related to both target and kinematics of movement [2], [15], [23]–[43]. Peri-movement activity, i.e., the activity around the time of movement, in the primary motor cortex, posterior parietal cortex (PPC), and dorsal premotor cortex (PMd) is related to the movement kinematics such as direction, velocity, position, and acceleration [2], [23]–[32]. In addition to perimovement activity, neural activity in the PPC has been shown to encode the intended target [15], [33]–[36] prior to movement initiation. Similar activity has also been observed in the premotor cortex including PMd [28], [30], [37]–[43]. Second, this approach more closely mirrors the natural way in which the sensorimotor system decides on a plan of action and executes its movement. In other words, the several components of the musculo-skeletal system are coordinated in order to reach a target and hence the target of a movement and the desired trajectory to reach it are strongly correlated [44], [45]. Indeed, there has been a body of offline work demonstrating the advantage of combining both target and trajectory related information in the decoder using either simulated neural data in [46]–[48] and in our work [49], [50], or previously recorded neural data in [11], [51]. We also presented promising results of a real-time BMI that jointly decodes the target and trajectory in [52]–[54].

In addition to modeling both the target and trajectory information, similar to the natural sensorimotor system, a BMI system can be modeled as a feedback control system. When using a BMI, the subject (controller) decides on the control commands and consequently modulates the neural activity to move the prosthetic device to a desired target (i.e., achieve the task goal) while receiving real-time visual feedback of the state of the movement. Based on these considerations, a more principled BMI design that aims to mirror the sensorimotor control system and jointly decode the movement target and the corresponding trajectory would allow for a potentially more accurate movement execution.

Here we develop a real-time BMI that uses a novel optimal feedback control design and combines information about target and trajectory intent, and demonstrate its implementation in sensorimotor tasks performed by two rhesus monkeys. This BMI employs a novel two-stage approach. In the first stage, it uses the neural spiking activity prior to movement initiation to predict the intended target of the movement. In the second stage, it combines this prediction with the peri-movement spiking activity to estimate the movement trajectory. To decode the trajectory, inspired by the optimal feedback control theory of the sensorimotor system [44], [45], [55]–[57], we build an optimal feedback-controlled state-space model for goal-directed movements, which we use in a recursive Bayesian decoder. We have derived and presented the algorithmic details of this decoder, used in the second stage of the BMI, in [49], [50] in a simulation study and assuming knowledge of the intended target, and in [58]. We implement the combined two-stage BMI for decoding movements in an instructed-delay center-out task. The two-stage BMI processes the spikes directly in real time, i.e., at the millisecond time-scale on which the neural spiking activity is recorded. Here, we show that the two-stage BMI performs better than either stage alone, demonstrating the advantage of combining both target and trajectory related information in real time. The optimal feedback control design results in trajectories that are smoother, have lower estimation error, and acquire the target more accurately. As a baseline, we also make offline comparisons to a linear ridge regression decoder [11], [19], a regularized variant of the commonly used linear least squares regression decoder [2]–[5], [7], on the training sessions (manual control) data and show that the two-stage decoder outperforms the regression decoder.

## Results

**Figure 1:**
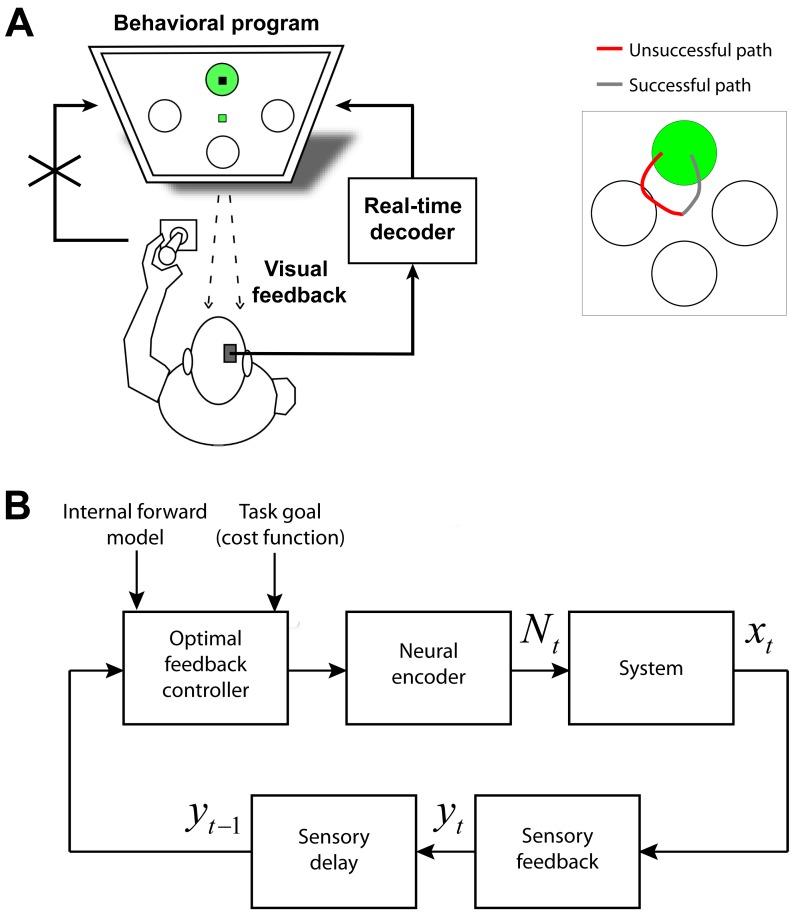
Experimental task and the optimal feedback control model. (A) Experimental task. The experiment consisted of an instructed-delay center-out task with four targets (*left*). To be rewarded, the monkey not only had to acquire the correct target, but also had to avoid touching any of the incorrect targets first (example successful and unsuccessful paths are shown on the *right*). After target presentation, there was 1 s of delay before the “go” cue, signaling that the monkey could begin moving the joystick. During the training sessions, the monkey controlled the position of the cursor using a joystick. During the BMI sessions, the joystick was disconnected and the real-time decoder controlled the cursor. (B) Optimal feedback control framework to model the BMI. An optimal feedback control framework is used to model the BMI motor task. In this framework, each task is performed to accomplish a goal during which there is real-time sensory feedback (e.g., visual feedback), 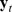, about the state of the system to be controlled (e.g., BMI), 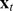. Based on the intended goal, the internal forward model about the dynamics of the system, and the sensory feedback about the state of the system, the brain (controller) decides on a control command, which is reflected in its neural activity, 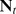, and controls the system (see Materials and Methods). Here we have assumed that the brain receives perfect sensory feedback of the state, i.e., 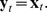

We measured the performance of our BMI in an instructed-delay center-out directional task in which two monkeys used a joystick to move a cursor from the center of the screen to one of four targets displayed at its periphery (see Materials and Methods). Unlike many BMI motor tasks in which the subject can freely move until reaching a target, this task required the monkey to reach the correct target *without* touching any of the incorrect targets under a limited time constraint. Hence only trajectories that reached the correct target and at no point touched an incorrect target placed at the other three sides of the screen were rewarded (Figure 1A). A performance measure used in these experiments was the acquisition accuracy, which is the percentage of trials on which the task is successfully completed. Multi-electrode spiking activity was recorded from PMd and the supplementary motor area (SMA) from which 20±2 neurons (mean ± s.d.) were isolated and used. At the beginning of each day, the monkey first performed the standard task using a joystick (training session) during which target and kinematic neural models were constructed. The monkey then performed the same task as before but this time cursor position was controlled by the neural activity recorded from the monkey (BMI sessions; Figure 1A) and the monkey received visual feedback of the cursor on the screen.

### Jointly Decoding the Target and Trajectory Using an Optimal Feedback Control Design

The BMI processing consisted of two stages and decoded two aspects of movement. During the first stage, it used a maximum-likelihood (ML) decoder based on a point process model of the neural spiking activity to predict the monkey's intended target of movement during the delay period after target presentation but before movement initiation (see Materials and Methods). In the second stage, the BMI combined this decoded target with the peri-movement activity to estimate the trajectory. In this stage, the spiking activity of each neuron was modeled as a point process fitted to position and velocity. The BMI estimated the trajectory using an optimal feedback control design that combined the decoded target with the peri-movement activity. This design is inspired by the optimal feedback control theory of the sensorimotor control system used to explain its function [44], [45], [55]–[57], [59], and is cognizant of the fact that in the BMI context the system to be controlled is the BMI instead of the musculo-skeletal system (Figure 1B; see Materials and Methods for details). The result is a recursive Bayesian decoder that we term the feedback-controlled parallel point process filter (FC-P-PPF). We introduced this decoder in [49], [50] assuming knowledge of the target and using simulated neural spiking activity. Here we implement this decoder as the second stage in the two-stage real-time BMI and show its performance in combination with target prediction and using neural recordings in instructed-delay center-out tasks both offline and in real time. In the BMI experiments, the decoder updated the estimated position of the cursor in fine-scaled steps of 5 ms in real time, which was also used to bin the spikes.

### Model Training and Validation

Models for the BMI were trained on the neural spiking activity during the training session at the beginning of each day. Neural recordings were made during target presentation prior to the presentation of the “go” cue, which signaled that the monkey could move the joystick, as well as during movement itself after the “go” cue (Figure 1A).

Each training session consisted of an average of 89±2 trials. Point process models relating the spiking activity of each recorded neuron to target location and movement kinematics were constructed based on the known target location and cursor position for each trial and the recorded multiple-neuronal activity using the generalized linear models (GLM) framework [60]. Models were then cross-validated (leave-one-out) on the same data by finding the corresponding target predictions and kinematic estimates. Target location was predicted using the ML decoder from the neural spiking activity in the 800 ms delay period prior to the “go” cue. Kinematics were estimated using either the two-stage decoder that combined the target predictions from the first stage with the peri-movement activity, or its second stage but not taking into account the target predicted from the first stage.

During the delay period, the ensemble spiking activity (20±2 neurons) predicted the correct target with high accuracy in the training sessions (leave-one-out cross-validation). The prediction accuracy of the trained point process target models across sessions (see Materials and Methods), measured as the percentage of trials on which the models correctly predicted the target using the delay neural activity, was 81±3% (mean ± s.d.). To examine further the contribution of the individual neurons to the target prediction accuracy, we performed a neuron dropping analysis in which the spiking activity of a single neuron during the delay period was used to decode the target (Figure S1A–D). We found that across sessions, 48±12% of the neurons had a target prediction accuracy significantly greater than chance (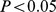). We further found that relatively few neurons (on average 3.3±1.0 across sessions) were sufficient to obtain a target prediction accuracy that was higher than 90% that of the ensemble (Figure S1E).

During movement, the premotor neurons were tuned to position and velocity. Fitting the point process models for the kinematics using the GLM framework [60] (see Materials and Methods), we found that across sessions 47±16% of the premotor neurons were significantly tuned to either position or velocity at least in one dimension (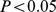; Bonferroni correction for multiple comparisons). Of these neurons, 57% were tuned to position only, 15% were tuned to velocity only, and 28% were tuned to both position and velocity. In agreement with previous studies, these findings suggested that the recorded premotor neurons held significant information about both the target and kinematics of the movement.

### Offline Model Comparisons

We tested the performance of the two-stage decoder in an offline analysis of the training sessions data using leave-one-out cross-validation. We also compared to the performance of the second stage of the decoder alone by replacing the feedback-controlled state-space model in the FC-P-PPF (see Materials and Methods) with a random-walk (RW) model, which uses no prior target information and only enforces smoothness in the trajectory. The resulting filter is the RW-PPF (see Materials and Methods). We also compared the performance of the two-stage decoder to that of the linear ridge regression decoder [11], [19], [61] that is a regularized variant of the commonly used least-squares linear regression decoder [2]–[5], [7] (see Materials and Methods). For each decoder we updated the position estimate every 5 ms, which was also the bin width for the spiking activity. Note that the chance level acquisition accuracy in our task using any decoder is at most 25% since there are four targets on the screen and hitting the wrong one results in an error. We also confirmed that estimating the trajectory from shuffled neural activity using a regression decoder results in approximately this chance level accuracy (see Materials and Methods).

**Figure 2:**
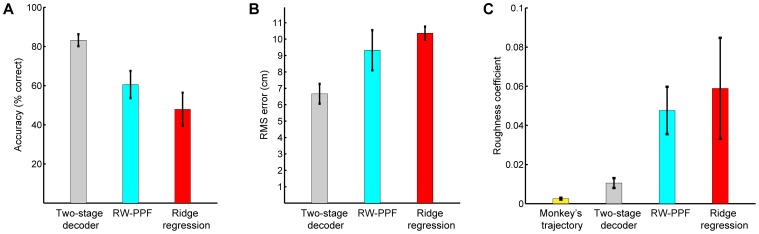
Offline model comparisons. The bars show mean quantities and the error bars show the standard deviation (s.d.) around the mean across sessions. All quantities are obtained from the training sessions using leave-one-out cross-validation. (A) Accuracy of the different models. (B) RMS error of the different models. (C) Roughness coefficient of the different models. The two-stage decoder (used in the real-time BMI) outperforms all other models in terms of accuracy, RMS error, and smoothness.

**Figure 3:**
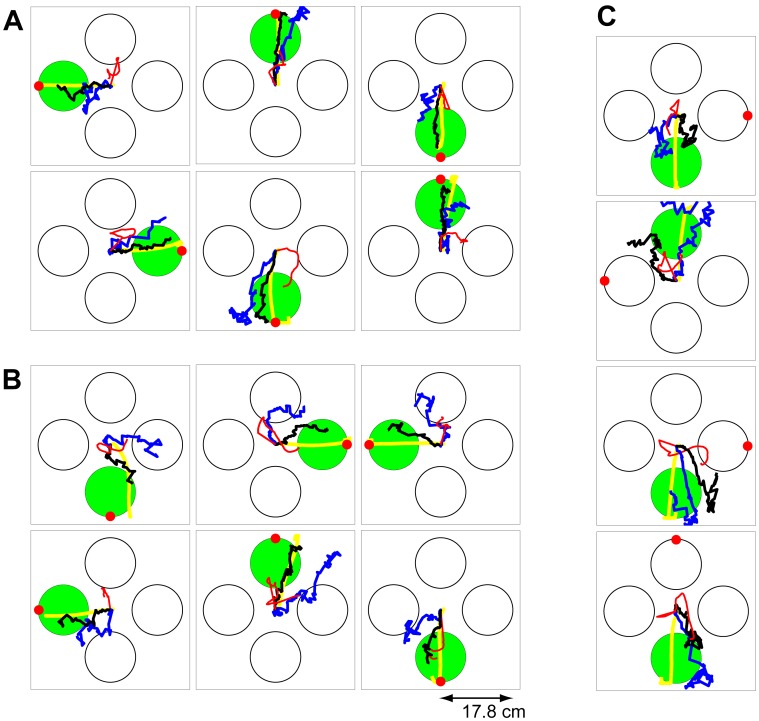
Comparison of the offline trajectory estimates. The green circle shows the instructed target and the yellow line shows the monkey's trajectory. The black line shows the trajectory estimate using the two-stage decoder, the red circle shows the predicted target from the first stage, the blue line shows the trajectory estimate of RW-PPF (i.e., the second stage of the decoder without using the target prediction), and the red line shows that of the linear ridge regression decoder. (A) Sample trials in which both the two-stage decoder and RW-PPF acquire the target correctly. (B) Sample trials in which the two-stage decoder acquires the target correctly but RW-PPF does not. (C) Sample trials in which the two-stage decoder acquires the target correctly but the target is predicted incorrectly from the first stage.

**Figure 4:**
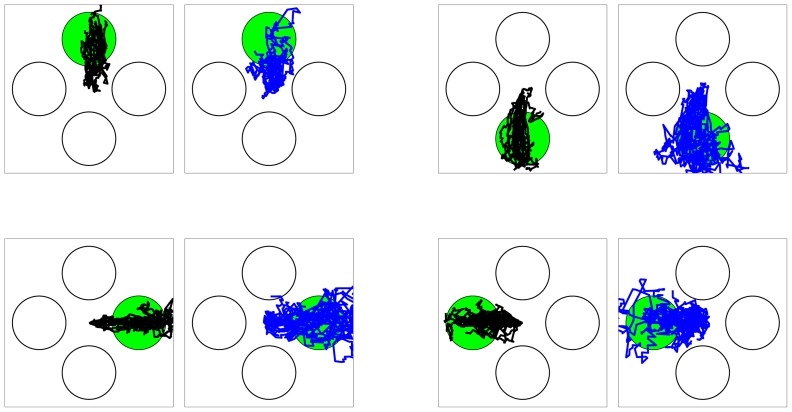
Trajectory estimates and their variations. The green circle shows the instructed target. The black lines show the trajectories estimated using the two-stage decoder in multiple trials in which the decoder is correct. The blue lines show the trajectories estimated by RW-PPF (i.e., the second stage of the decoder without using the target prediction) in multiple trials in which RW-PPF is correct.

We found that the acquisition accuracy of the two-stage decoder across sessions was 83±3%, higher compared to 61±7% for the RW-PPF that used only the peri-movement activity (one-sided McNemar test, 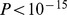; Figure 2A). Hence target predictions from the first stage of the decoder resulted in a correction rate of 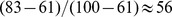
% for the inaccurate trajectory estimation in the second stage (Figure 3B). More specifically, while 15% of the trials with correct RW-PPF performance were not acquired correctly by the two-stage decoder, 80% of the trials with incorrect RW-PPF performance were corrected in the two-stage decoder, resulting in an overall improvement. Also, the trajectories estimated by the two-stage decoder were closer to the monkey's trajectory (Figure 3A,B and Figure 4). To quantify this, we measured the average root mean-square (RMS) error across all trials (Figure 2B). We found that the RMS error of the RW-PPF on average was 40% higher than the two-stage decoder (one-sided *t*-test, 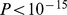). As an alternative measure of error between the estimated and the true trajectories, we also computed the signal-to-noise ratio (SNR) that is calculated as the ratio of the desired signal (joystick position) variance and the mean-squared estimation error, 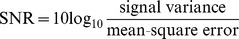, and is used in a number of BMI studies [13], [62], [63]. We found, consistently, that the SNR of RW-PPF was lower than the two-stage decoder (one-sided *t*-test, 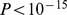; Figure S2). While first-stage target prediction alone does not estimate a continuous trajectory, we can compare it to the two-stage decoder in terms of target acquisition accuracy. Making this comparison, we found that the second stage resulted in a correction rate of 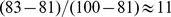
% for the target prediction errors (one-sided McNemar test, 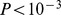; Figure 3C; Figure S3). Here, only 0.2% of the trials with correct target prediction were not acquired correctly by the two-stage decoder, while 12% of the trials with incorrect target prediction were corrected in the two-stage decoder. However, as these were offline estimation of fast joystick movements, the more appropriate test for the correction of the incorrect target predictions by the peri-movement activity in the second stage is in real-time BMI sessions (see below).

We also compared the performance of the two-stage decoder to that of a linear ridge regression decoder (Figure 3). The ridge regression decoder reconstructed the position at each time as a linear function of the history of the ensemble firing rates, which were calculated every 5 ms in sliding bins of 100 ms (see Materials and Methods for more detail). We included up to 800 ms—same duration as the delay period used for target prediction—of history coefficients in the regression decoder. Specifically, we found the performance of the ridge regression decoder using 200 ms, 400 ms, 600 ms, and 800 ms of history coefficients and selected the number of history coefficients that minimized the mean-square error using leave-one-out cross-validation. The average accuracy of the ridge regression decoder across sessions was 48 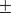 8%, which was lower than the two-stage decoder (one-sided McNemar test, 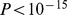; Figure 2A). Also the average RMS error of the ridge regression decoder was 55% higher than the two-stage decoder (one-sided *t*-test, 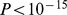; Figure 2B). Consistently, the SNR of the ridge regression decoder was lower than the two-stage decoder (one-sided *t*-test, 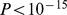; Figure S2). Note that the ridge regression decoder uses the delay period activity due to its history coefficients.

Finally the trajectory estimations in the two-stage decoder were smoother than either the RW-PPF or the ridge regression decoder (Figure 3). To quantify this, we calculated the average roughness coefficient [64] (see Materials and Methods) for each of the decoders (Figure 2C). The roughness coefficient measures the degree of smoothness in the estimated trajectory and is smaller for smoother estimates. We found that the average roughness coefficients of the RW-PPF and the linear ridge regression decoder were 4.5 and 5.6 times larger than that of the two-stage decoder, respectively (one-sided *t*-test, 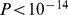 in both cases).

### Combined Target and Trajectory Decoding in a Real-Time BMI

**Figure 5:**
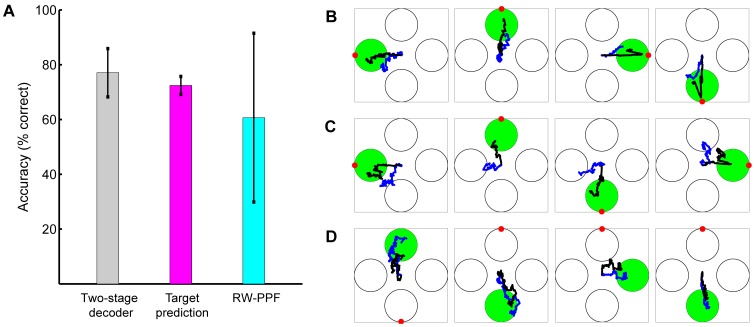
Real-time trajectories and the complementary property of the two-stage BMI. (A) Comparison of the real-time BMI accuracy with the real-time target prediction accuracy from the first stage and also with the accuracy of RW-PPF (i.e., the second stage without using the target prediction) obtained offline using the same real-time data set. The bars show mean quantities and the error bars show the standard deviation (s.d.) around the mean across sessions. The two-stage BMI outperforms either stage alone. (B–C) Sample decoded trajectories. In all panels, the green circle shows the instructed target, the red circle shows the predicted target from the first stage, the black line shows the trajectory estimate of the two-stage BMI, and the blue line shows that of RW-PPF using only the peri-movement activity. Sample trials where both the two-stage BMI and RW-PPF correctly acquire the target are shown in B. The complementary property of the two-stage BMI is illustrated in C and D. C shows sample trials in which the correct target prediction of the first stage compensates for the inaccurate estimation of the kinematic decoder in the second stage. D shows sample trials in which the kinematic decoder in the second stage, using ongoing peri-movement activity, compensates for the incorrect target prediction of the first stage.

To investigate whether kinematic and target related activity can be jointly used to obtain accurate motor performance in real time, monkeys performed the same task as before but using a BMI. The task was again a center-out task requiring the monkey to move the cursor using the BMI to the correct target without touching any of the incorrect targets. The real-time BMI used the two-stage decoder. During the 800 ms delay period prior to the “go” cue, the BMI predicted the target and after the “go” cue it combined this target information with the peri-movement activity using the optimal feedback control design (FC-P-PPF) to decode the trajectory. Note that the cursor was held at the center during the delay period. We found that using the two-stage BMI, the monkeys could perform the task with an average accuracy of 77±9% (Figure 5A). To assess the stability of the performance throughout the recordings per day, we compared it in the first and second half of sessions. We found that accuracy did not change significantly (Wilcoxon rank-sum test, 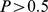) and remained stable.

We found that the two stages of the BMI performed in a complementary manner to achieve its overall accuracy; the correct target predictions could compensate for the inaccurate performance of the kinematics decoder and the ongoing trajectory estimation could correct the incorrect target predictions. To illustrate this complementary property of the BMI and how its two stages contributed to its overall performance, we compared with the performance of each of its stages alone using either the target-related delay or the kinematic-related peri-movement activities. To compare the accuracy of the BMI with that of using only the peri-movement activity, i.e., using the second stage of the BMI alone without target information, we decoded the trajectory offline using RW-PPF on the same real-time data set (Figure 5B–D). The average acquisition accuracy of the RW-PPF was 61
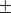
31% (Figure 5A). This suggests that the first stage of the BMI resulted in a correction rate of 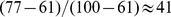
% for the second stage errors (Figure 5A,C). More specifically, while 14% of the trials with correct RW-PPF performance were not acquired correctly by the BMI, 63% of the trials with incorrect RW-PPF performance were acquired correctly by the BMI, resulting in an overall improvement. While the first stage of the BMI alone (i.e., real-time target prediction) cannot estimate the continuous trajectory, we can still compare it to the two-stage BMI in terms of target acquisition accuracy. The average real-time target prediction accuracy of the first stage of the BMI was 72
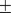
3%. This indicates that the second stage of the BMI (FC-P-PPF) resulted in a correction rate of 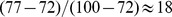
% for the target prediction errors of the first stage (Figure 5A,D). More specifically, while 4% of the trials with correct target prediction were not acquired correctly by the BMI, 27% of the trials with incorrect target prediction were acquired correctly by the BMI, resulting in an overall improvement. Hence the joint performance of the BMI was higher than either stage alone using either kinematic or target related activity. Also the trajectories estimated by the BMI were smoother than those of the RW-PPF and had a significantly lower roughness coefficient.

The acquisition time in the BMI sessions, i.e., time until the trial ended by rewarding the monkey, was close to the natural acquisition time in the training sessions. In our experiments we used a short 3 s time-out condition to make the task more challenging and the required acquisition time closer to that of monkey's own movement. The median acquisition time for the natural movement was 0.6
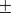
0.3 s and for the two-stage BMI was 0.9
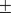
0.5 s.

### Control Comparisons

To determine differences in BMI performance across monkeys and therefore the robustness of the two-stage BMI to individual variability, we examined differences in performances between the two monkeys, A and B. For monkey A, the real-time BMI accuracy (percentage of trials in which the correct target was acquired without the cursor touching the incorrect targets) was 67
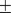
4%. Comparing to the second stage alone using only the peri-movement activity, we found that the accuracy of the RW-PPF on the real-time data set for this monkey was 29
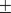
1%. Hence, even though the kinematic tuning in this monkey was weak, the real-time BMI had a relative high accuracy. This showed that the first stage of the BMI resulted in a correction rate of 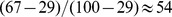
% for the inaccurate performance of the trajectory decoder. The real-time target prediction accuracy of the first stage using only the delay activity in this monkey was 71
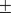
1%. Note that because of the weak kinematic tuning in the recorded neurons in this monkey, the correction happened only by the first stage. For monkey B, the real-time BMI accuracy was 82
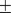
5%. Comparing to the second stage alone using only the peri-movement activity, we found that the accuracy of the RW-PPF in this monkey was 76
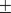
24% and hence the first stage of the BMI resulted in a correction rate of 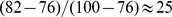
% for the inaccurate trajectory estimation of the second stage. Comparing to the average real-time target prediction accuracy of the first stage, which was 73
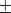
4%, suggested that the second stage of the BMI in monkey B resulted in a correction rate of 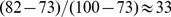
% for the target prediction errors in the first stage. Therefore, in this monkey both stages exhibited the corrective behavior.

Together these findings suggest that although the performance of one stage in the two-stage BMI may not be equal across monkeys or recording areas, by combining both target and trajectory related information the two-stage approach provides a robust computational system that maintains good accuracy under variable experimental conditions (see Figure S4 for a comment on ridge regression).

## Discussion

Based on our understanding of the sensorimotor system [44], [45], [55]–[57], [59], natural movement incorporates information about the intended target as well as the trajectory of the movement. We implemented a novel real-time BMI designed to mimic the sensorimotor control system by a two-stage approach: First the activity prior to movement initiation is used to predict the intended target, and second this prediction is combined with the peri-movement spiking activity to estimate the trajectory using an optimal feedback control design. This is to our knowledge the first time that sensorimotor optimal feedback control principles have been used to decode the intended movement from neural recordings. It is also the first time that combined estimation of target and trajectory has been done in real time.

To decode the movement, we used an optimal feedback control design inspired by the optimal feedback control theory of the sensorimotor system [44], [45]. In this view, each task is performed to accomplish a goal during which there is sensory feedback about the external state of the system. Specifying an approximate forward dynamics model, modeling the real-time sensory feedback about the state of the system, and quantifying the task goals as cost functions and the desired time to accomplish them, we can predict the next plan of action or control command by finding the one that minimizes the cost function. For a BMI, the same framework can be applied to predict the next plan of action. The difference is that the system being controlled is the BMI instead of an individual's own musculo-skeletal system. Hence the individual's next plan of action is reflected in the neural activity, this activity controls the BMI, and the individual in turn receives real-time visual feedback of the resulting movement (Figure 1B). Also, the BMI does not have knowledge of the desired time to accomplish the goal, which is decided by the controller (the individual). The present BMI hence resolved this movement duration uncertainty based on the neural spiking activity in real time (see Materials and Methods; [49], [50]). The BMI also processed the spiking activity directly and hence operated at the millisecond time-scale of the spikes. In addition to its application to interpreting the sensorimotor function, optimal feedback control has also been deemed valuable for interpreting the neural basis of movement in the motor cortical areas [65]. This further motivates the use of optimal feedback control principles for the design of real-time BMIs.

In addition to modeling the BMI as an optimal feedback control system, we also decoded both target-related delay and kinematic-related peri-movement activities using a two-stage design. We demonstrated that the two stages in the BMI functioned in a complementary manner. When the spiking activity for one stage was less informative, the other stage often provided sufficient information for the BMI to reach the correct target. As a result, the two-stage BMI performed better than either stage alone. Overall, the estimated trajectories using the two-stage optimal feedback control design were more accurate, had lower RMS error, and were smoother than the linear ridge regression decoder or a random-walk point-process decoder.

Unlike “free-roaming” motor tasks in which subjects could move freely until reaching a target, the present task was demanding in that at no point the trajectories could touch the incorrect targets and then proceed to the correct target. This was considered an incorrect response (Figure 1A). In addition the response time was constrained. Despite this, the monkeys were able to achieve a relatively high accuracy using the two-stage BMI (77
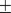
9%). This accuracy was obtained by using relatively few neurons (11 on average) that were tuned to either target or trajectory.

Our BMI used direct point process modeling of the spiking activity. It hence processed the spikes directly in real time as opposed to a rate function calculated from these spikes as is done in previous real-time BMI work. Recent work [66] has demonstrated that reducing the bin width used to calculate the firing rates of the spiking activity, which are in turn used as input in a BMI, improves its performance. An interesting question for future investigation in real-time experiments is therefore whether moving to the time-scale on which the spiking activity is recorded, i.e., processing the spikes directly, could improve the performance of real-time BMIs.

The two-stage decoder combined both the target-related activity prior to movement and the trajectory-related peri-movement activity in real time. This combination makes the two-stage decoder robust to variations across recording conditions. Hence in scenarios where one type of activity is not strongly tuned in a recording area, it could still be possible for the decoder to result in acceptable performance. For example, if the delay activity is not strongly tuned to the target in an experiment, the second stage, i.e., the feedback-controlled parallel point process filter, can still be used to model the BMI target-directed movement and decode it from the peri-movement activity. In this case the decoder would need to additionally put a prior distribution on the target locations since no target information can be obtained prior to movement (see Materials and Methods and [51]). It is also important to note that in certain applications, such as key selection, there may be no need for estimating the continuous trajectory, and faster performance may take precedence over more accurate performance. In such scenarios, first-stage target prediction alone using the delay activity may be a valuable approach [15], [16].

In the two-stage BMI, one stage compensated for the inaccuracies of the other and vice versa. Since our model relies on neural activity to estimate the movement, we cannot test directly to what extent the brain uses these two aspects of motor control to execute a movement. It is interesting to speculate, however, that similar to findings made in these experiments, the premotor cortex may use information on intended target location to correct for discrepancies in ongoing movement. Similarly, it may use information about ongoing movement to fine-tune differences between initially intended target location and target location during movement itself. Hence, in addition to enabling the design of more accurate decoding algorithms, the present optimal feedback control design may provide insight into the control aspects of the motor function in natural settings. Therefore the two-stage optimal feedback control based BMI could provide an important and unique new step in developing neuroprosthetics that take advantage of the multiple types of movement information.

## Materials and Methods

### Ethics Statement

This study was performed in strict accordance with the recommendations in the Guide for the Care and Use of Laboratory Animals of the National Institutes of Health, and under regulation of the Subcommittee on Research Animal Care at Harvard Medical School. The protocol was approved by the Institutional Animal Care and Use Committee for Massachusetts General Hospital (animal welfare assurance number: A3596-01). All surgery was performed under inhalational anesthesia in combination with Ketamine and opiate analgesia, and every effort was made to minimize suffering, in accordance with the recommendations of the Weatherall report, “The use of non-human primates in research”. Animals were housed in their cages in the primate animal facility. Animals were fed regularly with regulated fluid administration and were given environmental enrichment. Animals were completely unrestrained within their home cages. At the conclusion of experiments one animal was euthanized to allow for histological verification of recording sites with Ketamine and Pentobarbital, in conformance with recommendations of the American Veterinary Medical Association (AVMA) Guidelines on Euthanasia.

### Behavioral Task

We used two adult male rhesus monkeys (*macaca mulatta*) in the study. During the tasks, the animals were seated in a primate chair (Crist Instrument Co Ltd, Damascus, MD). The primates' head were restrained using a head post, and a spout was placed in front of their mouth to deliver juice using an automated solenoid. A spring-loaded, two-degrees of freedom manipulandum was mounted anterior to chair on the side contralateral to recording. A computer monitor was placed in front of the animals at eye level which displayed the task. A NI DAQ card (National Instruments, TX) was used for the I/O behavioral interface, and the behavioral program was run in Matlab (MathWorks, MA) using custom made software (www.monkeylogic.net).

Primates performed a center-out visually-instructed motor directional task that penalized touching the incorrect targets. The monkeys held the joystick contralateral to the site of recordings and could move their limb freely in the horizontal and vertical dimensions during the task. A computer monitor displayed the target locations and a cursor was used to represent the position of the joystick handle. Each individual trial began with the presentation of a central fixation point surrounded by four gray circular targets. Once the animals held the cursor within a central radius for a delay of 500 ms, one of the four randomly selected targets would turn green. After another 1000 ms, the fixation point would change color (“go” cue), at which time the monkeys could use the joystick to move the cursor from the center of the screen to the instructed target. Once the cursor reached the target, the animal received a drop of juice following a 320 ms delay if the correct target was reached and no incorrect targets were touched by the cursor. If during a trial, the animals moved prematurely, failed to reach any target during the allowed time or touched an incorrect target before reaching the correct target, the trial aborted. Note that all targets had the same size as illustrated in Figures 3, 4, and 5. Once reward was delivered, another 1000 ms would lapse, the targets would erase, and the sequence would repeat again. The animals were required to return the spring-loaded joystick to the center fixation point before a new trial began.

### Neurophysiologic Recordings and BMI Setup

A titanium head post and recording electrodes were surgically implanted in each monkey contralateral to the side of joystick use. All procedures were performed in an IACUC-approved aseptic primate surgical facility. Prior to electrode implantation, craniotomies were performed over the sites of interest using standard stereotactic coordinates. Once the cortex was exposed and the sulcal anatomy identified, several silicone microelectrode arrays were placed in the cortex (Neuronexus technologies, MI) into the PMd and supplementary motor areas. The electrodes were secured into place using fibrin glue, silicone sealant, and methylmethacrolate. The distal leads were then attached to a female connector and secured to the skull with titanium miniplates and dental acrylic. Anatomic post-mortem confirmation of electrode positioning was performed in one monkey. The second monkey is still performing behavioral tasks.

Recordings began at two weeks following surgical recovery. A Plexon multichannel acquisition processor was used to amplify and band-pass filter the neuronal signals (150 Hz
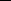
8 kHz; Plexon Inc., TX). Shielded cabling carried the signals from the electrode array to a set of six 16-channel amplifiers. Signals were then digitized at 40 kHz and processed to extract action potentials in real time by the Plexon workstation. Classification of the action potential waveforms were accomplished using dual-window discrimination and principle component analysis. Units with stable, identifiable waveform shapes and adequate refractory periods determined by autocorrelation were then used for the real-time experiments. Joystick position was sampled and recorded at 1 kHz. Neuronal data obtained from the Plexon workstation, in the form of action potential time stamps and channel, were then transmitted to a second PC computer running Matlab (Mathworks, MA) in real time. On decoder training sessions, the primates would use the joystick to move a cursor on the screen to one of four randomly selected targets over multiple trials. On decoder BMI sessions, the monkeys would still be allowed to use the joystick but the cursor image displayed on the screen would be supplied by the Matlab real-time decoder (Figure 1A). The cursor was initially placed at the center fixation point at the beginning of each trial. Here, estimated cursor movements would be relayed through a DAQ I/O (National Instruments, TX) to a third PC computer running the behavioral task. The computer would then display the estimated cursor position.

### Chance Level Accuracy

Since the task requires reaching the correct target and since *first* touching *any* of the other (incorrect) targets results in an error, at best any chance-based decoder would *first* reach the correct target with equal probability among the four possible. Hence the performance of such a chance-based decoder is at most 25%. We also examined the performance of a linear regression decoder on shuffled neural activity and confirmed that it approximately resulted in 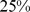 accuracy. To do so we shuffled the calculated firing rates for each neuron across time and trials, keeping its average firing rate the same.

### Target Decoding

The BMI decodes the monkeys' intended target of movement by recording the ensemble spiking activity during the 800 ms delay interval prior to the “go” cue. Note that the delay between the start of target presentation and “go” cue is 1000 ms. We do not use the activity in the first 200 ms in the BMI in order to allow sufficient time for the visual target information to reach the PMd and SMA [16]. Using offline cross-validation analyses we observed that discarding this activity improves the prediction accuracy. Spiking activity of each neuron during this delay interval is modeled as a homogeneous Poisson process (a point process with constant rate) whose firing rate is a function of the intended target, fitted using the GLM framework. A maximum-likelihood (ML) decoder first calculates the likelihood probability of this ensemble activity for each possible target, 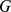, and then selects the target with the highest likelihood as its prediction. Denoting the neural point process observations of the ensemble of 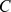 neurons by 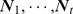 where 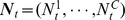 is the binary spike events of the 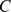 neurons at time 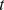, and assuming that the neurons are conditionally independent given the target, the point process likelihood model for the ensemble is given by [60]

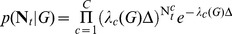
(1)where 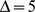 ms is the time increment used for binning the spikes and 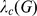 is the modeled firing rate of the 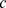
th neuron during the delay period for target 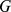. The ML decoder then predicts the target as the one maximizing the ensemble likelihood,



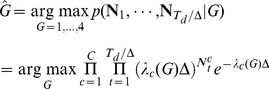
(2)Here 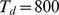 ms is the delay period. During the delay period the cursor was held at the center of the screen.

### Kinematic Decoding

In the second stage, the BMI combines the decoded target with the peri-movement ensemble spiking activity using a recursive Bayesian decoder with an optimal feedback control design. A recursive Bayesian decoder in general consists of two probabilistic models: the prior model on the time sequence of kinematic states, and the observation model relating the neural signal to these states. The prior model should in general incorporate any prior information available about the kinematic states, which for a goal-directed movement includes the intended target. We build the prior movement model of the decoder using an optimal feedback control design, which takes into account the decoded target location and the sensory feedback. To develop the observation model, we use a point process model of the spiking activity whose instantaneous rate is a log-linear function of kinematics. The resulting decoder hence processes the spikes directly in real time and operates at the millisecond time scale of the spiking activity. In the next few sections we present the prior movement model and the neural observation model used in the decoder for estimation of goal-directed movements, and the decoder recursions.

### Goal-Directed Movement Model: an Optimal Feedback Control Design

Previous offline work have built goal-directed prior models for reaching movements by conditioning a linear Gaussian state-space model, also known as a random-walk model, on being at the target at a *known* arrival time [46], [67] or using a linear feedforward controlled (i.e., not taking into account the sensory feedback) model again assuming a known arrival time [47]. Alternatively, goal-directed prior models have been built by using a training data set, for example fitting a linear Gaussian state-space model for a given target to empirical reaches to its location [51] or fitting a single model for arbitrary targets based on a data set of reaches to their locations [11].

Since the goal of the decoder is to estimate the intended movement of the subject, a prior movement model that aims to emulate the sensorimotor processing underlying motor control could result in more accurate estimation of movement. Hence we build the prior goal-directed state-space model for the movement kinematics based on the optimal feedback control theory of the sensorimotor system [44], [45]. This theory has been successfully used to interpret the sensorimotor function. For example, it has been shown that this theory can predict the bell-shaped velocity profiles and straight line trajectories observed in reaching movements [56], [57]. In this optimal feedback control framework, each task is performed to accomplish a goal during which there is sensory feedback about the state of the system. Based on the intended goal, the system's forward dynamics model, the sensory feedback (for example vision and proprioception) about the current state of the system, and the desired time to accomplish the goal, the subject (controller) decides on the next plan of action or control command (for example muscle activation) and can hence make real-time adjustments based on the feedback to improve behavior. Specifying an approximate forward dynamics model and quantifying the task goals as cost functions and also the sensory feedback, this framework can predict the next plan of action in the presence of model uncertainty and sensory noise.

The difference in applying this framework to natural arm movement [44], [45] and movement using a BMI is that in the latter case the system to be controlled is the BMI (Figure 1B). While performing the BMI task, the monkey decides on the next control command based on the visual feedback of the cursor position and the intended target. Similar to natural movement, in movement using a BMI, the next plan of action is in turn reflected in the neural activity but this time controls the system or BMI through the decoder (as we will develop) as opposed to directly controlling the arm (Figure 1B). Hence the BMI can be modeled in this optimal feedback control framework.

Motivated by this view, we develop a prior feedback-controlled state-space model for the kinematics that exploits the information about the target location and takes into account the sensory feedback. We introduced this model in [49], [50] in a simulation study. Based on this model, the decoder can predict the monkey's next plan of action or control command and consequently the next kinematic state. Note that our prior model does not rely on a training data set, as is the case in [11], [51], and can therefore easily extend to different target locations without requiring a set of empirical reaches to these locations. Finally, by using the optimal feedback control formulation, it could generalize to tasks other than reaching movements, if desired, by simply quantifying the goals of such tasks as the cost function in this formulation. We now present the construction of this model.

Denoting the sequence of kinematic states by 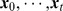, we assume they are generated according to the linear dynamical system,
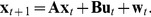
(3)This is the forward dynamics model. Here, 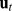 is the control signal at time 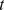, which is decided by the controller (the primate in the BMI context), 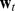 is the zero-mean white Gaussian state noise with covariance matrix **W**, and **A** and **B** are parameters of the forward model. Here we assume that the sensory feedback 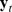, is noiseless and 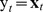. This means that we assume the monkey has perfect sensory feedback of the cursor position on the screen. We also implicitly assume that the brain has acquired an internal representation of this forward model, i.e., has formed an internal forward model of the dynamics of movement in response to control commands 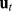 in the task [59]. To find 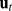 in the control framework, we need to specify a cost function that will then be minimized by optimizing over 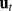. The cost function in a given task should quantify its goal. For the above linear Gaussian dynamics, if we pick the cost function as a quadratic function of the state and control variables, i.e.,
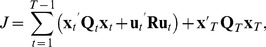
(4)where *T* is the movement duration, 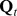 is positive semidefinite and **R** is positive definite, then the optimal control signal at any time, 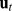, is simply a linear *feedback* of the state at that time [68], i.e.,
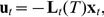
(5)where 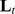 can be found recursively and offline [68]. This is the well-known linear quadratic Gaussian (LQG) solution. Note that we assumed the monkey has perfect visual feedback of the cursor state, 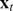, in BMI control. Hence this optimal feedback control policy can be interpreted as the monkey's corrective control command in response to the visual feedback of the BMI cursor state that may deviate from the intended trajectory.

Substituting (5) in (3) reduces this state-space model to the optimal feedback-controlled state-space model
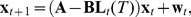
(6)which can now be used as the prior model to make prediction on the kinematic states. Note that 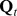 and **R** should be appropriately designed for an application of interest and 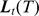 is time-varying and a function of the duration *T*. Note also that the sensory feedback is incorporated in the control term since the control term is simply a linear function of the current kinematic state (see (5)), assumed to be known through the feedback. This is in turn reflected in the prior model in (6).

We can now specialize these to the reaching movements used in our experiments. For a reaching movement the cost function should enforce end-point positional accuracy, stopping condition, and energetic efficiency [56], [57]. Denoting the desired target position by **d** ^*^ and taking the state to be 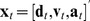 where the components represent position, velocity and force in the two dimensions respectively, similar to previous studies [56], [57] we take this cost function to be the weighted sum
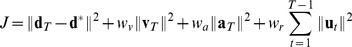
(7)where the weights are chosen to penalize the terms in the cost function approximately equally on average [56], [57]. We adapt the following first order lowpass muscle-like system [57] for the dynamical system in (3) in each dimension,
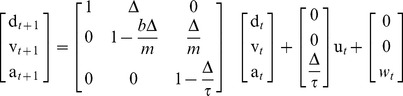
(8)where the parameters 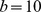 Ns/m, 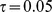 s, and 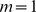 kg come from biomechanics [57]. Note that we again assume that the monkey has acquired an internal forward model of the dynamics of movement in the task consistent with the above forward model [59] and uses this internal forward model to decide on the next control command after switching from manual control to biomimetic BMI control. The noise term in the forward model 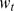 in turn captures the uncertainty in the forward model. In general, we can allow for this internal forward model to change once in BMI mode especially if a non-biomimetic decoder is used in combination with learning. Such considerations, however, are outside the scope of this paper.

Having specified the forward dynamics model and the cost function for the reaching movement, the feedback matrices 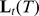 can now be easily precomputed offline from the recursive solution of LQG [68] and stored for real-time use. Having these matrices, we can predict the monkey's next plan of action reflected in the control signal, 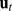, using (5) and assuming perfect sensory feedback about the current state of the cursor on the screen, 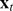. Note that in our two-stage decoder 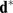 is determined from the decoded target location in the first stage (for more algorithmic details see [49], [50], [58]).

### Observation Model

We build the observation model for each neuron as a point process whose instantaneous firing rate is a function of kinematics [60], i.e.,
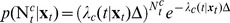



We used a modified version of the cosine tuning model [23], [26] for the instantaneous firing rate, modeling it as a log-linear function of position and velocity in the two dimensions [60], i.e.,
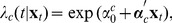
(9)where 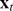 denotes these kinematic states at time *t* and 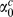 and 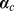 are fitted using the GLM framework [60] on the peri-movement spiking activity. More specifically, denoting the model parameters for neuron 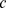 by 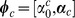, the GLM framework finds the maximum likelihood estimate



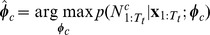
where 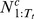 is the peri-movement spiking activity of the neuron during training and 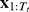 are the corresponding kinematic states. Using the GLM framework, *P*-values can also be obtained for all the model parameters [60] (for example using the glmfit function in Matlab) and hence the tuning properties of the neurons can be examined.

We assumed that the spiking activity of the neurons are conditionally independent given the kinematic states and hence the observation model for the ensemble is given by
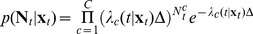
(10)


### Uncertainty in the Movement Duration

Having the prior and the observation models we can now develop the recursions for the Bayesian decoder. However, the prior model built in (6) is dependent on the movement duration, 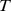, which is not known to the decoder. In other words, unlike natural movement in which the monkey (controller) decides on the movement duration, in movement using a BMI the decoder does not have a priori knowledge of this duration. This is typically the case for goal-directed state-space models as there is much more constraint on the movement kinematics close to the arrival time at the target compared to far from it since in the former case the trajectory soon needs to reach the intended target [49], [50], [58]. Hence we develop the BMI decoder to jointly resolve this duration uncertainty and estimate the trajectory purely based on the neural spiking activity. We first present the recursions of a feedback-controlled point-process filter assuming a known movement duration and then show how we can resolve the duration uncertainty inherent to the prior model.

### Feedback-Controlled Point Process Filter (FC-PPF) for a Known Movement Duration

For now we assume that the movement duration is known. The minimum mean-square error (MMSE) estimator is given by the mean of the posterior density that is 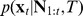 for a given duration 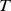. Denoting the one step prediction mean by 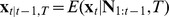, its covariance matrix by 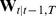, the MMSE estimate by 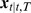, and finally its covariance matrix by 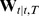, 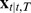 is found from the following recursions
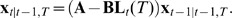
(11)

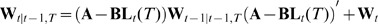
(12)

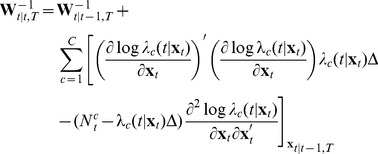
(13)

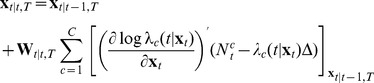
(14)where 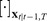 denotes the evaluation of the expression at 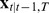. These recursions are obtained using (6) for the prediction step and a Gaussian approximation for the update step as previously derived [49], [50], [69]. For the rate model in (9) since 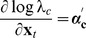 and 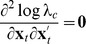 these recursions simplify to



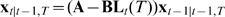
(15)

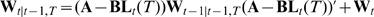
(16)

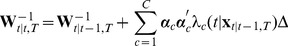
(17)

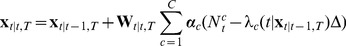
(18)


To provide some insight into these recursions, note that in the prediction step given in (15) the feedback-controlled prior model is used to move the estimate forward. In the update step given in (18) the estimate is found by making a correction or update to this prediction. Here, 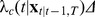 is the predicted probability of having a spike in the time interval 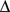 and hence the correction is (1- predicted probability of a spike) if a spike occurs and (0- predicted probability of a spike) if no spike occurs. Hence if a spike occurs and the predicted probability of a spike is high this correction is small and vice versa. Therefore the estimate is a combination of the prediction and the correction terms. The more informative the spiking activity is about the state (determined through 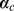), the more weight is placed on the correction term and vice versa. If the spiking activity is not informative at all or is not used, then the estimate will just be the prediction, which is obtained only using the feedback-controlled state model and ignoring the observation model. In this case and given a movement duration, the prediction step will generate a straight line from the center to the predicted target location according to the prior model.

### Resolving the Duration Uncertainty: Feedback-Controlled Parallel Point Process Filter (FC-P-PPF)

The feedback-controlled state-space model in (6) (and many other goal-directed state-space models) is a function of movement duration, *T*, not known a priori to the real-time BMI [49], [50], [58]. Hence any goal-directed real-time decoder needs to resolve this duration uncertainty. We introduced a framework to resolve this duration uncertainty in [49] by discretizing the movement duration, finding the kinematic estimate for each discretized duration, and then optimally combining these kinematic estimates based on the neural data. A similar approach using a discretized set of durations was subsequently used in a simulation study in [70] to resolve the duration uncertainty of the prior model developed in [46] for estimation of simulated trajectories. Our framework for resolving the duration uncertainty is based on mixture modeling, a common approach in statistical inference that is used to estimate a desired density in different applications. For example, mixture modeling combined with sequential state estimation in dynamical systems, when the system is operating under different or changing regimes of operation, has been used as early as in [71]. See also the mixture Kalman filtering work in [72] and references therein. For decoding the kinematics from neural activity, mixture modeling was first used in [51] and successfully applied to combine empirically fitted and time-invariant state models for reaching movements to different targets in an offline study. Here, we use mixture modeling to combine feedback-controlled prior models of different durations and hence resolve the duration uncertainty inherent to this prior model.

Our framework works by discretizing the duration, finding the kinematic estimate for each discretized duration using an FC-PPF, running these FC-PPF filters in parallel, and finding the likelihood of each of the discretized duration points jointly with the corresponding trajectory estimate, all purely based on the neural spiking activity and in real time. The decoder then optimally combines the trajectory estimates corresponding to the discretized durations to get the overall trajectory estimate. The result is the feedback-controlled parallel point process filter (FC-P-PPF) that we have derived in detail in [49], [50], [58]. Denoting the overall MMSE estimator by 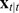, it is given by the mean of the posterior density, which using the law of total probability is expanded as,
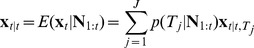
(19)where 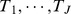 are the 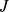 discretization points for 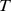, 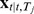 is the estimate given that the duration is 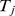 and found from the recursions in (15) to (18), 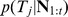 is the likelihood of the corresponding duration given the peri-movement neural activity, and finally the summation is over all 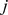 for which 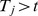. The likelihood of the corresponding duration, 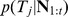, can be computed as derived in our work in [49], [50], [58] and is only a function of the prediction and posterior means and covariances found in (15) to (18) and the parameters of the observation model. Here we provide the final expressions for readers' convenience. These are given by
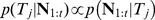
(20)with



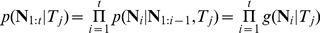
(21)Using a Gaussian approximation to the posterior, the term 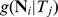 as we derive in [49], [50], [58] is given by
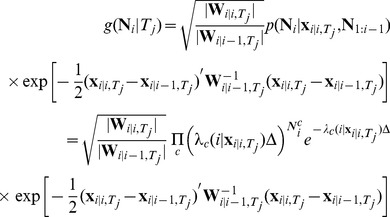
(22)for 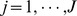 where all the quantities are known. Note that 
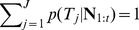
 and hence we can compute all the duration likelihoods. Finally, combining in (19), we get the final estimate. Here, we coarsely discretized the duration of a trial.

### Random-Walk Point Process Filter (RW-PPF)

Setting **B** = **0** reduces the state-space model in (3) to the random-walk model and consequently the recursions of FC-PPF with this choice recover the RW-PPF.

### Ridge Regression

We also find the performance of the linear ridge regression decoder on the neural data. The commonly used regression decoder in the BMI literature is the ordinary linear least squares regression decoder that is fit by minimizing the residual squared error. The linear least squares regression, however, can result in high mean-square estimation error due to a high error variance, for example in cases with correlated regressors [61]. An alternative to the ordinary linear least squares regression is the linear ridge regression that can result in lower mean-square error by reducing the error variance at the price of some increase in bias [61]. Hence ridge regression has been used in a number of BMI studies [11], [19]. In ridge regression, the position variable at a given time is reconstructed as a linear combination of the history of the standardized firing rates of the ensemble of 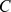 neurons over a selected number of time bins prior to and including that time. In ridge regression, the regression coefficients are found by minimizing the residual squared error plus a regularization term that penalizes large coefficient estimates [11], [61]. Denoting the total number of time samples in the training data set by 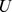, the number of history coefficients in the regression model by 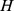, the 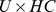 dimensional standardized firing rate matrix with **R**, the mean subtracted position variable by 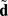, and the regression coefficients by 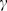, the ridge regression coefficients are given by [11], [61].
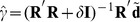
(23)where 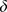 is the regularization parameter. The special case of 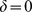 gives the ordinary least squares regression solution. We selected 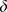 by finding the mean-square error for a wide range of regularization parameters and selecting the regularization parameter that minimized the mean-square error using leave-one-out cross-validation. We found the performance using multiple choices for the length of the history window used by the ridge regression. Specifically, we found the performance of the ridge regression decoder using 200 ms, 400 ms, 600 ms, and 800 ms of history coefficients and selected the number of history coefficients that minimized the mean-square error using leave-one-out cross-validation.

### Possible Extensions

In our implementation of the BMI, the first stage of the decoder makes a decision about the intended target. However, instead, the BMI can easily include all targets in the FC-P-PPF but weight them properly by their corresponding likelihood calculated from the first stage similar to a previous offline study [51]. In our case, this means including more parallel filters in the FC-P-PPF for the different targets (and their discretized durations). This will consequently increase the complexity of the decoder. We chose not to implement this extension since, using offline analysis, we observed that it resulted in little improvement at the price of four times the complexity. The absence of a significant improvement in this case was likely a result of the fact that in our experiments the target-related activity during the delay period was strongly tuned to the targets and that overall the peri-movement activity was not as strongly tuned to the task. However, in cases where such target-related activity is not strong, this extension will potentially result in further improvement in the second stage as it allows for its higher weighting compared to the first stage. Also applying such an extension could potentially allow the FC-P-PPF decoding algorithm to be applied in situations where no delay period activity and hence no target information from the first stage is available. In such a case, equal weights would be initially assigned to the filters corresponding to each possible target in the FC-P-PPF, and these weights would then be updated during movement based on the peri-movement activity.

Even though in the present experiments we used four targets, the decoder can generalize to the case with more targets. This is because the prior model in (6) can be generalized to arbitrary target locations by just replacing **d** ^*^ accordingly in the cost function in (7). Given the present results, it is conceivable that a similar complementary performance could be observed for the two-stage BMI in the case where more targets are present, especially given that in this case it will be harder for either stage alone to result in accurate performance (for example the first stage needs to decode one out of more targets). Investigating the behavior of the decoder in experimental setups with more target locations will be a valuable future research direction.

Finally, in our work we use the target onset and “go” cues to indicate the boundaries between baseline, plan, and movement epochs as is typically done in BMI experiments [73]. For a BMI to be truly autonomous, however, such epochs should also be detected based on the neural activity (see, e.g., [73]). Hence extending our approach to also detect the movement epoch based on the neural activity will be a future research direction.

### Number of Neurons Required for Accurate Target Prediction

To find the number of neurons that were sufficient to obtain an accurate target prediction during the delay period, we performed a single neuron analysis in which the spiking activity of a single neuron was used to decode the target (Figure S1). We then sorted the neurons based on their single-neuron accuracies. From the sorted set, we selected different number of neurons and performed the decoding analysis for them. For example the decoding analysis for two neurons was done for the two neurons with the highest single neuron accuracies. Doing so, we found the target prediction accuracy as a function of the number of top cells included in the decoding. We found that on average across sessions only 17
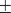
6% of the neural ensemble or 3.3
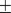
1.0 neurons were sufficient to obtain a prediction accuracy higher than 90% of the ensemble accuracy.

### Roughness Coefficient

The roughness coefficient for a sequence is an indicator of how smooth it is [64]. For a sequence 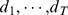, it is defined as
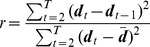
where 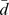 denotes the mean of the sequence. It can also be generalized to the vector case by writing



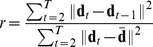



For a trajectory of duration 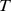 this provides a measure of smoothness where smaller coefficients correspond to smoother trajectories. In this case 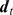 corresponds to the position vector at time 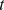.

## Supporting Information

Figure S1 **Neuron dropping analysis.** (A–D) Activity of a single neuron under the four targets in one training session. In the top figure, each row corresponds to a different trial and the black dots indicate the spike times. The bottom figure shows the corresponding firing rate. Activity is aligned to the target presentation time and vertical dashed lines indicate the target presentation and “go” cue times. The target prediction accuracy (leave-one-out cross-validation) of this neuron is 65%. (E) Target prediction accuracy as a function of the number of top neurons. The solid lines show the target prediction accuracy of six training sessions as a function of the number of neurons included in the prediction (The curve for each session is shown in a different color for clarity). Neurons were sorted based on their single neuron accuracy. Chance level accuracy for target classification is 25%.
(TIF)

Figure S2 **Offline SNR comparisons.** The bars show mean quantities and the error bars show the standard deviation (s.d.) around the mean across sessions. SNR is obtained from the training sessions using leave-one-out cross-validation.
(TIF)

Figure S3 **Offline accuracy of the two-stage decoder on the training sessions data.** Accuracies of the two-stage decoder, the first stage target prediction, and the RW-PPF (i.e., the second stage without using the target prediction) on the training sessions joystick movements are shown. The bars show mean quantities and the error bars show the standard deviation around the mean across sessions. Comparisons of the RMS error and the smoothness of the decoded trajectories for the two-stage decoder and RW-PPF are given in Figure 2. Note that target prediction, unlike the two-stage, RW-PPF, and the linear regression decoders, does not generate an estimated trajectory.
(TIF)

Figure S4 **Comparison of the trajectory estimates.** For completeness, we also found the accuracy of the ridge regression decoder on the same real-time BMI data set. It is important to note, however, that since the performance of the ridge regression here is found offline, it is likely lower from its performance if used in real time and practiced by the monkey. The black line shows the trajectory estimate of the real-time BMI. The blue line shows that of RW-PPF using only the peri-movement activity, and the red line shows that of a linear ridge regression decoder, both run offline but using the same real-time data set as the BMI. (A) Sample trials in which the ridge regression decoder is correct. (B) Sample trials in which the ridge regression decoder is incorrect. The average accuracy of the linear ridge regression decoder on the BMI data sets was 50±11% (mean ± s.d; c.f. Figure 5A).
(TIF)
