# Supplementary figures and images for: A Real-Time Brain-Machine Interface Combining Motor Target and Trajectory Intent Using an Optimal Feedback Control Design
Source: PLoS One. 2013 Apr 10;8(4):e59049. doi: 10.1371/journal.pone.0059049 (PMC3622681; doi:10.1371/journal.pone.0059049)

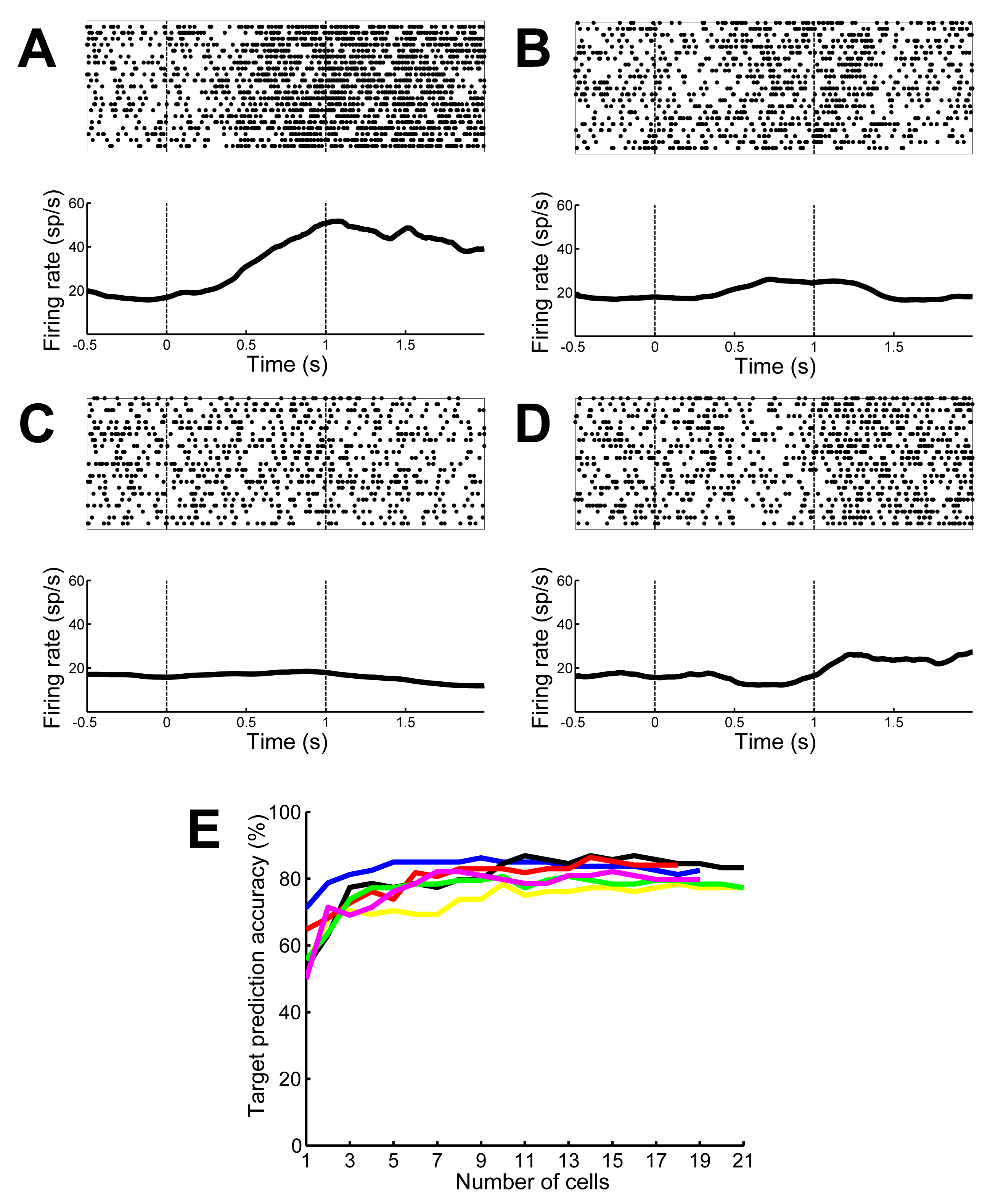

Supplement: Figure S1 — Neuron dropping analysis. (A–D) Activity of a single neuron under the four targets in one training session. In the top figure, each row corresponds to a different trial and the black dots indicate the spike times. The bottom figure shows the corresponding firing rate. Activity is aligned to the target presentation time and vertical dashed lines indicate the target presentation and “go” cue times. The target prediction accuracy (leave-one-out cross-validation) of this neuron is 65%. (E) Target prediction accuracy as a function of the number of top neurons. The solid lines show the target prediction accuracy of six training sessions as a function of the number of neurons included in the prediction (The curve for each session is shown in a different color for clarity). Neurons were sorted based on their single neuron accuracy. Chance level accuracy for target classification is 25%. (TIF) [file pone.0059049.s001.tif]

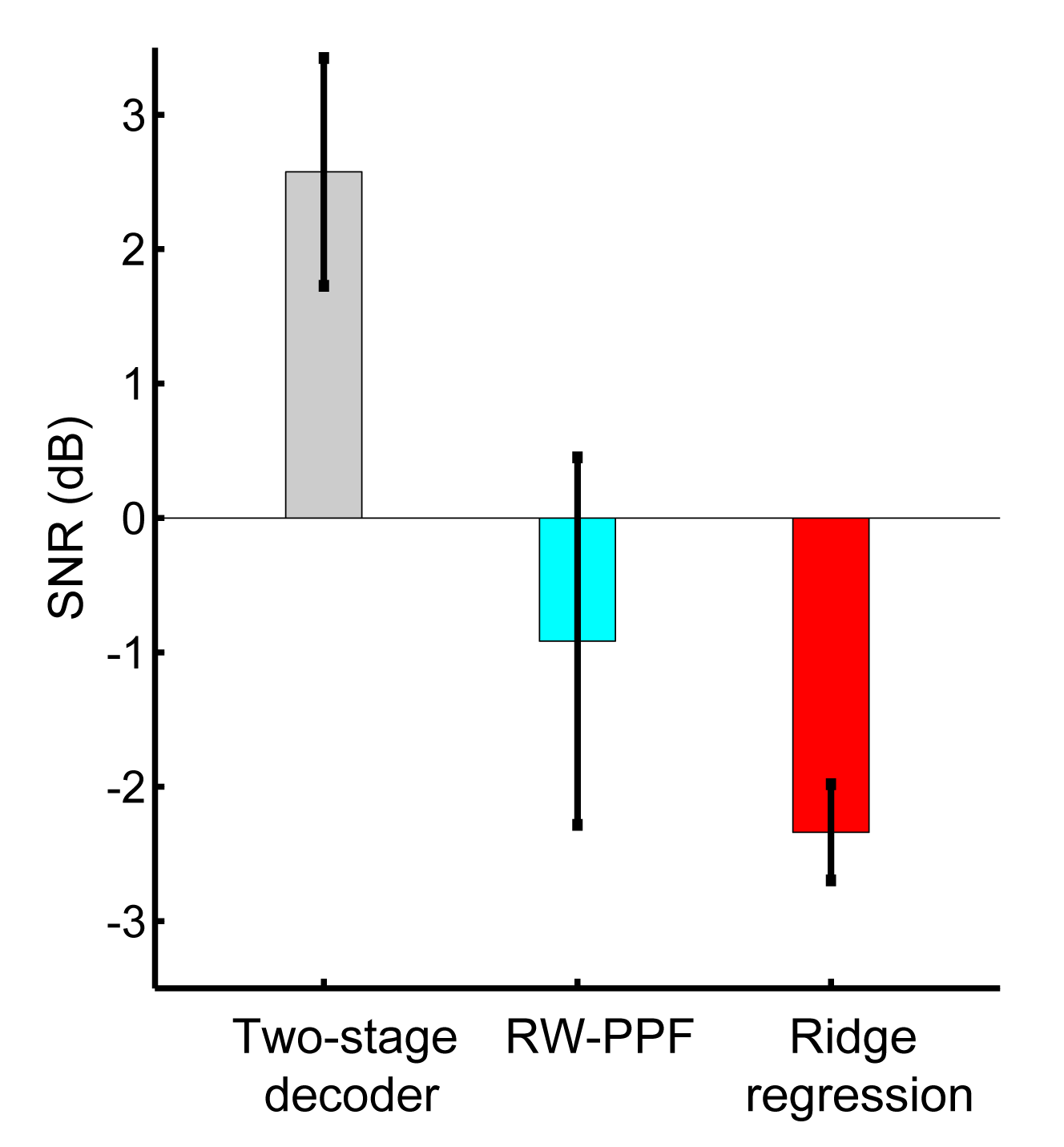

Supplement: Figure S2 — Offline SNR comparisons. The bars show mean quantities and the error bars show the standard deviation (s.d.) around the mean across sessions. SNR is obtained from the training sessions using leave-one-out cross-validation. (TIF) [file pone.0059049.s002.tif]

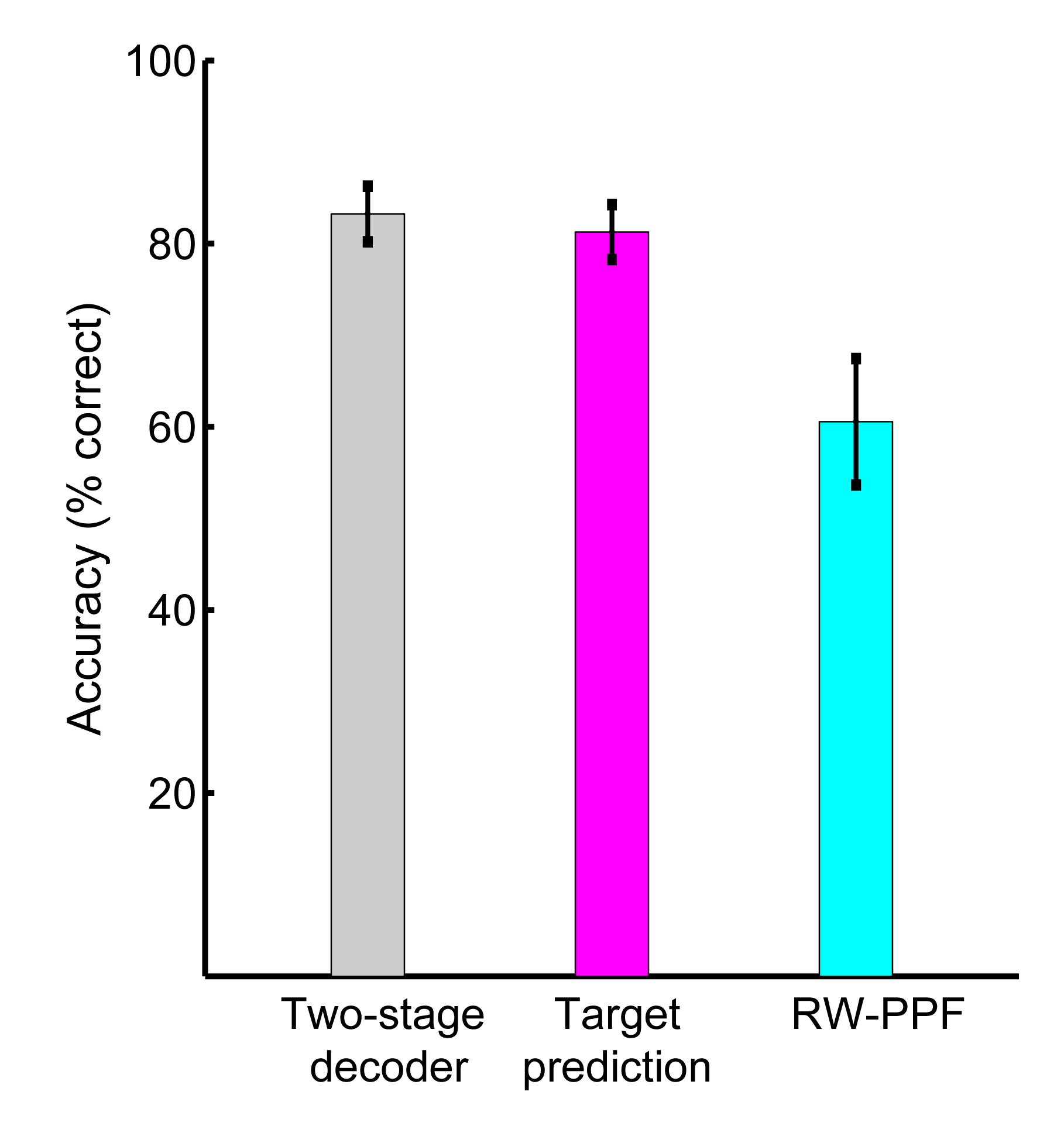

Supplement: Figure S3 — Offline accuracy of the two-stage decoder on the training sessions data. Accuracies of the two-stage decoder, the first stage target prediction, and the RW-PPF (i.e., the second stage without using the target prediction) on the training sessions joystick movements are shown. The bars show mean quantities and the error bars show the standard deviation around the mean across sessions. Comparisons of the RMS error and the smoothness of the decoded trajectories for the two-stage decoder and RW-PPF are given in Figure 2. Note that target prediction, unlike the two-stage, RW-PPF, and the linear regression decoders, does not generate an estimated trajectory. (TIF) [file pone.0059049.s003.tif]

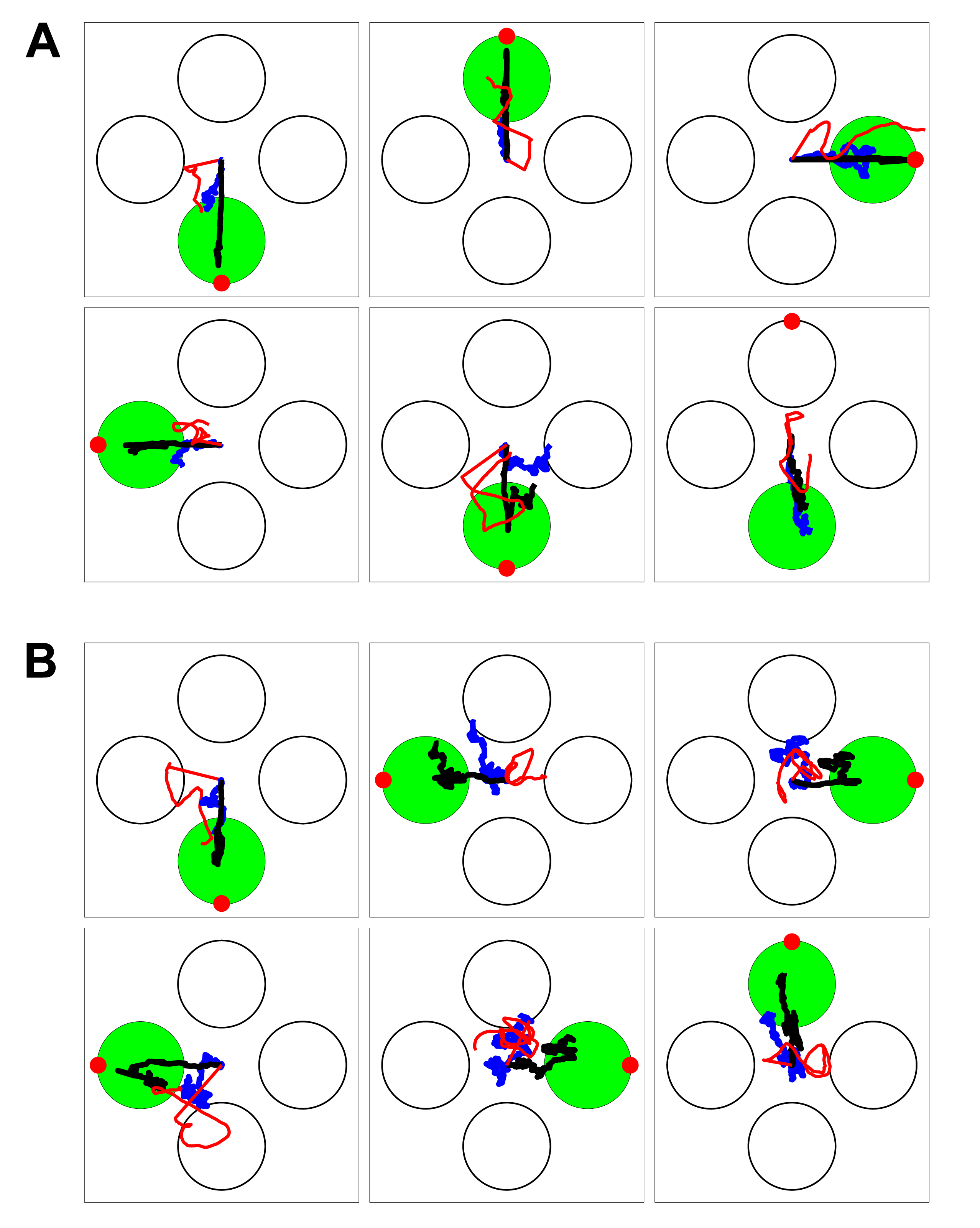

Supplement: Figure S4 — Comparison of the trajectory estimates. For completeness, we also found the accuracy of the ridge regression decoder on the same real-time BMI data set. It is important to note, however, that since the performance of the ridge regression here is found offline, it is likely lower from its performance if used in real time and practiced by the monkey. The black line shows the trajectory estimate of the real-time BMI. The blue line shows that of RW-PPF using only the peri-movement activity, and the red line shows that of a linear ridge regression decoder, both run offline but using the same real-time data set as the BMI. (A) Sample trials in which the ridge regression decoder is correct. (B) Sample trials in which the ridge regression decoder is incorrect. The average accuracy of the linear ridge regression decoder on the BMI data sets was 50±11% (mean ± s.d; c.f. Figure 5A). (TIF) [file pone.0059049.s004.tif]
